# Construction and validation of programmed cell death-based molecular clusters for prognostic and therapeutic significance of clear cell renal cell carcinoma

**DOI:** 10.1016/j.heliyon.2023.e15693

**Published:** 2023-05-02

**Authors:** Yanlin Tang, Changzheng Zhang, Chujin Ye, Kaiwen Tian, Jiayi Zeng, Shouyu Cheng, Weinan Zeng, Bowen Yang, Yanjun Liu, Yuming Yu

**Affiliations:** a Department of Urology, Guangdong Provincial People's Hospital (Guangdong Academy of Medical Sciences), Southern Medical University, Guangzhou, China; b Shantou University Medical College, Shantou, China; c The Second School of Clinical Medicine, Southern Medical University, Guangzhou, China; d School of Medicine, South China University of Technology, Guangzhou, China; e Guangdong Cardiovascular Institute, Guangdong Provincial People's Hospital Guangdong Academy of Medical Sciences, Guangzhou, China; f Department of Immunology, School of Basic Medical Science, Southern Medical University, Guangzhou, China

**Keywords:** Clear cell renal cell carcinoma, Programmed cell death, Gene expression, Tumor microenvironment, Immunotherapy

## Abstract

As the dominant histological subtype of kidney cancer, clear cell renal cell carcinoma (ccRCC) poorly responds to conventional chemotherapy and radiotherapy. Although novel immunotherapies such as immune checkpoint inhibitors could have a durable effect in treating ccRCC patients, the limited availability of dependable biomarkers has restricted their application in clinic. In the study of carcinogenesis and cancer therapies, there has been a recent emphasis on researching programmed cell death (PCD). In the current study, we discovered the enriched and prognostic PCD in ccRCC utilizing gene set enrichment analysis (GSEA) and investigate the functional status of ccRCC patients with different PCD risks. Then, genes related to PCD that had prognostic value in ccRCC were identified for the conduction of non-negative matrix factorization to cluster ccRCC patients. Next, the tumor microenvironment, immunogenicity, and therapeutic response in different molecular clusters were analyzed. Among PCD, apoptosis and pyroptosis were enriched in ccRCC and correlated with prognosis. Patients with high PCD levels were related to poor prognosis and a rich but suppressive immune microenvironment. PCD-based molecular clusters were identified to differentiate the clinical status and prognosis of ccRCC. Moreover, the molecular cluster with high PCD levels may correlate with high immunogenicity and a favorable therapeutic response to ccRCC. Furthermore, a simplified PCD-based gene classifier was established to facilitate clinical application and used transcriptome sequencing data from clinical ccRCC samples to validate the applicability of the gene classifier. We thoroughly extended the understanding of PCD in ccRCC and constructed a PCD-based gene classifier for differentiation of the prognosis and therapeutic efficacy in ccRCC.

## 1 Introduction

Kidney and renal pelvis cancers are now among the 10 most commonly diagnosed cancers in both sexes, and 79,000 new cases were estimated to be identified in 2022 in the United States [1]. Of this type of cancer, about 85% are renal cell carcinoma (RCC), whose principal histological subtype is clear cell (ccRCC) [2]. Although the incidence rate has been steady, the death rate has decreased in recent years [3]. This phenomenon could be partly due to the development of novel immunotherapy, among which immune checkpoint inhibitors (ICIs) were the most popular. Drugs such as Nivolumab and Pembrolizumab have been proven to exert enduring effects on responders and listed as the first-line therapies for ccRCC [4,5]. However, it is difficult to identify the responders while the resistance remains unpredictable even in the responders [6]. To deal with these problems, substantial effort has focused on discovering therapeutic biomarkers for ICIs and the expression level of some related molecules was frequently utilized in the clinic. For example, high PD-L1 expression may be more frequently identified in patients with better response toward ICIs. Nonetheless, the measurement technique and cutoff value for PD-L1 expression have not been unified and those with low PD-L1 expression could also have considerable responses to ICIs [7,8]. Therefore, the discovery of novel reliable biomarkers is required for the individualized application of ICIs in ccRCC.

The therapeutic function of ICIs could be influenced by the tumor microenvironment (TME) in each patient [9]. As one of the major components of TME, the infiltration of immune cells was shown to be associated with the ICIs response. Patients with a high density of infiltrated cytotoxic lymphocytes respond better toward PD1/PD-L1 blockade [10]. Meanwhile, different subtypes and functional statuses of the infiltrated lymphocytes could also influence the therapeutic effect of ICIs [11,12]. Another influencing factor that attracted wide attention is the neoantigen load. More neoantigens may be correlated with a higher probability of recognizing the tumor cells and eliciting an immune response [13]. In recent years, various measures, including but not limited to tumor mutation burden (TMB) have been constructed to reflect the neoantigens load and used as therapeutic biomarkers for ICIs [14,15]. With these concerns, a comprehensive understanding of the TME and immunogenicity in ccRCC could facilitate taking full advantage of the therapeutic effect of ICIs.

Programmed cell death (PCD) is an important mechanism for the host to erase damaged cells with tumor propensity and to maintain homeostasis. Even among neoplasms, PCD could also serve as a weapon for removing neoplastic cells [16]. With the further investigation in recent years, more and more subtypes of PCD were identified and their connectivity with TME gradually emerged [17]. Ferroptosis [18], pyroptosis [19], and necroptosis [20] were newly recognized as non-apoptotic PCDs with immunogenic features, being able to regulate the tumor immune microenvironment through releasing the cellular contents. Meanwhile, tumor treatments, including chemotherapy and radiotherapy, may exert their function by inducing PCD while some explanations for reduced drug efficacy may due to the developed apoptosis resistance in tumor cells [21]. This connection was also discovered while applying ICIs. Studies have shown that the induction of PCD, like pyroptosis, could improve the efficacy of ICIs [22,23], and the function of ICIs may achieve through PCD induced by CD8^+^ T cells [24,25]. Hence, a profound investigation of PCD in ccRCC could enhance our overall comprehension of the TME in ccRCC and assist in utilizing ICIs in clinic.

**Fig. 1:**
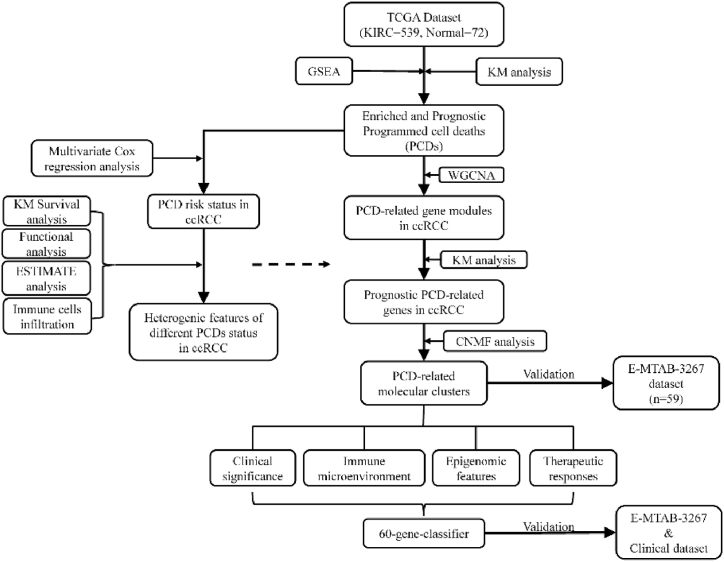
The flowchart of the construction of PCD-based molecular clusters.

In the current study, we made use of GSEA to discover the featured PCD in ccRCC and investigate the characteristics of different levels of PCD. With the help of Weight gene correlation network analysis (WGCNA) and non-negative matrix factorization (CNMF), cell death-related genes were identified and utilized to discover different molecular clusters of ccRCC patients whose TME features and clinical significance were distinct from each other. At last, a convenient gene classifier was built up for clinicians to predict the prognosis of ccRCC patients and prescribe individualized ICI-based therapies for them (Fig. 1).

## 2 Materials and methods

### 2.1 Data retrieval and gene sets enrichment analysis

Via Genomic Data Common (GDC) data portal (https://portal.gdc.cancer.gov/), RNA-sequencing data of ccRCC in KIRC project was extracted in the format of FPKM from The Cancer Genome Atlas (TCGA) together with the corresponding clinical data. 539 tumor samples and 72 normal samples were included in this data and the mRNA expression data were compared between these two groups using R package, ‘limma’ [26]. 5137 differentially expressed genes (DEGs) in ccRCC were identified with the threshold of both the absolute value of log Fold Change (logFC) > 1 and p-value <0.05. Besides, PCD-related keywords, including apoptosis, autophagy, ferroptosis, necroptosis and pyroptosis, were respectively searched in GSEA (http://www.gsea-msigdb.org/gsea/index.jsp) in which 5 annotated gene sets were acquired (KEGG_APOPTOSIS, WP_AUTOPHAGY, WP_FERROPTOSIS, GOBP_NECROPTOTIC_SIGNALING_PATHWAY, REACTOME_PYROPTOSIS). Then, we investigated the enrichment of these 5 PCDs in ccRCC by inputting the expression data, sample information from TCGA, and the gene sets into GSEA 4.1.0. During analysis, we regarded FDR q-value less than 0.05 as significant. Thereafter, 507 ccRCC samples were screened for the following analysis by excluding samples whose recorded survival time was less than 30 days.

### 2.2 Identification of prognostic PCDs in ccRCC

Single sample gene sets enrichment analysis (ssGSEA) serves as a method to obtain individual enrichment score of the targeted gene set for every single sample by calculating the gene expression data [27]. Achieved by R package ‘GSVA’ [28], the enrichment scores of the above 5 PCD-related gene sets for 507 ccRCC samples were calculated. Making use of these scores and the time information, the prognostic PCDs in ccRCC was obtained through Kaplan Meier survival analysis. After that, the prognostic PCDs were analyzed by multivariate cox regression analysis to establish a model for calculating PCD risk, PCD risk = coefficient A * score A + … + coefficient N * score N (A-N represent each PCD). In this way, each ccRCC samples obtained a PCD risk and the mean of these risk was used to separate high and low PCD risk groups.

### 2.3 Weight gene correlation network analysis (WGCNA)

To discover PCD-related genes in ccRCC, we introduced WGCNA, which could group interconnective genes into modules and correlate them with targeted features [29]. 5137 DEGs and the prognostic PCD mechanisms of 507 ccRCC samples were enrolled in the analysis. At first, the samples were clustered to recognize and exclude the outliers. Then, a topology overlap matrix (TOM) was transformed from the data matrix with an estimated soft threshold determined by scale independence as well as mean connectivity. Next, gene modules were derived from DEGs by calculating the dissimilarity of TOM and each module contained at least 30 genes as well as a dissimilarity of less than 0.25. After that, the module eigengenes were calculated and correlated with the prognostic PCDs to identify PCD-related gene modules.

### 2.4 Unsupervised clustering via consensus non-negative matrix factorization (CNMF)

DEGs in the PCD-related gene modules were extracted and Kaplan Meier analysis was conducted to deliver PCD-related prognostic DEGs with criteria of p-value less than 0.05 and 5-year survival difference more than 0.1. Then, these genes were incorporated into the CNMF algorithm to cluster the ccRCC samples. CNMF algorithm is a feasible access to reduce the dimension of large-scale gene expression data and recognize molecular patterns [30]. Employing the R package ‘CancerSubtypes’ [31], we respectively tested the results of 2, 3, and 4 clustering. The final clustering number was determined according to the calculated silhouette coefficients, which could assess the fitness of the clusters. On a scale of −1 to 1, a silhouette coefficient close to 1 means the cluster is notably distinguished from others. Besides, Kaplan Meier analysis aided in demonstrating the survival difference between the molecular clusters.

### 2.5 Analysis of the clinical characteristics in the molecular clusters

Clinical information in the TCGA cohort (age, gender, Fuhrman grade, AJCC stage, T stage, N stage, and M stage) was processed and arranged by the characteristics. After that, chi-square test was applied to compared these clinical features between the molecular clusters. At the same time, we applied univariate cox regression analysis and multivariate cox regression analysis in recognizing whether the molecular clusters as well as the clinical characteristics could predict ccRCC prognosis.

### 2.6 Investigation of the TME compositions in ccRCC

To gather an overview of the ccRCC TME, we introduced a scoring system named ESTIMATE (Estimation of STromal and Immune cells in MAlignant Tumors using Expression data), which could quantify the immune and stromal status and calculate an estimate score representing tumor purity [32]. In order to further investigate immune components, the infiltration levels of 23 types of immune cells were estimated for each ccRCC samples through ssGSEA algorithm based on a list of immune signatures [33]. Meanwhile, stroma cell enrichment data of KIRC project was acquired from xCell (https://xcell.ucsf.edu/), which utilizes both deconvolution and gene set enrichment methods [8]. Then, these data were separated in line with the molecular clusters and compared.

### 2.7 Analysis of the immunogenicity in ccRCC

Data of several immunogenic features, cancer testis antigens (CTA), homologous recombination deficiency (HRD), intratumoral heterogeneity (ITH), single-nucleotide variation (SNV), and TMB were acquired [34] and evaluated between different molecular clusters. A gene signature related to immune suppression was also collected to aid in understanding the immunogenicity through ‘GSVA’. Besides, Tumor Immune Dysfunction and Evasion (TIDE) helps calculated T-cell dysfunction scores according to the strategy provided by Jiang et al. [35]. If the T-cell dysfunction score of a gene was high, it may tend to counteract the survival benefit of cytotoxic T cell, suggesting its association with cytotoxic T cell dysfunction. Furthermore, we extracted the expression data of inhibitory immune checkpoints, immune chemokines and receptors as well as major histocompatibility complex (MHC), and analyzed their difference in different clusters. In addition, the mutation data of the ccRCC samples from TCGA was available in the public web tool cBioPortal (https://www.cbioportal.org/) and we investigated the mutation status in each molecular clusters through the R package ‘maftools’ [36].

### 2.8 Functional enrichment analysis

Gene ontology (GO) is widely known for providing comprehensive annotation of human biology on three levels, biological process, cellular component, and molecular function. R package ‘clusterProfiler’ [37] serves as an approach in the current study to investigate the enrichment functions in different clusters with the help of the GO database. Additionally, 10 pathways related to oncogenesis were retrieved [34] and investigated between clusters through ssGSEA.

### 2.9 Analysis of the therapeutic response

To further validate the clinical significance of the molecular clusters, we analyzed their property in differentiating therapeutic responses. TCIA (The Cancer Immunome Database, https://tcia.at/home) provides data on drug response toward immune checkpoint inhibitors (PD1, CTLA4, and PD1+CTLA4 inhibitors) through a kind of value called immunophenoscore deduced from integrated immunogenomic analysis [38]. Besides, the R package ‘pRRophetic’ [39] is able to utilize the trial data in GDSC (Genomics of Drug Sensitivity in Cancer, https://www.cancerrxgene.org/), calculating drug sensitivity for each sample. Axitinib, Pazopanib, Sorafenib, and Sunitinib were enrolled and analyzed between the molecular clusters. Furthermore, we analyze the response to the modulation of interferon-gamma (IFN-gamma) as well as tumor necrosis factor-beta (TGF-beta) signaling pathways [34].

### 2.10 Validation of the molecular cluster

An RNA expression dataset of ccRCC, E-MTAB-3267, which contains 59 tumor samples and the corresponding prognostic information, was acquired from ArrayExpress (https://www.ebi.ac.uk/arrayexpress/). The same prognostic PCD-related DEGs were used to cluster this cohort in the same way as mentioned above. For advanced clinical application, we constructed a gene classifier from the top 30 differentially expressed genes of each cluster for identification of the molecular clusters with the help of the nearest template prediction (NTP) algorithm which is a validated method to undergo class prediction through a group of signature genes [40]. By using the R package ‘CMScaller' [41], gene-based classes were predicted and validated in the TCGA and E-MTAB-3267 cohorts.

### 2.11 Transcriptome sequencing analysis

In order to provide additional evidence of the practical use of the gene-based classifier, we conducted transcriptome sequencing analysis of ccRCC and adjacent normal tissue collected from surgical resected samples. From June 2020 to July 2021, we collected 18 ccRCC and 6 normal samples from 6 ccRCC patients who were untreated before and underwent laparoscopic nephrectomy in Guangdong Provincial People’s Hospital. The resected tumor specimens from these patients were diagnosed as ccRCC through H&E staining and Immunohistochemistry staining by 2 independent pathologists from Guangdong Provincial People's Hospital (Table S1). The hospital ethics committee had approved our project with informed consent collected. According to the protocol provided by manufacturer, the NovaSeq 6000 high-throughput sequencing platform (Illumina, USA) was used for paired-end sequencing of each ccRCC and adjacent normal sample. After that, the sequencing reads that included aptamer sequences and low-quality reads and bases were removed Then, the high-quality pairwise reads that remained were aligned to the human genome GRCh38 using HISAT2 (v2.1.1), resulting in BAM files. The BAM files were organized using samtools (v1.15.1) and the reads were counted using Subread (v2.0.1). Fragments Per Kilobase of exon model per Million mapped fragments (FPKM) were converted from and used to substitute raw counts of transcripts per gene, allowing better comparability between samples at the transcriptome level. Thereafter, the genes used to build up the predictive classifier were differentially analyzed between the normal and ccRCC samples with the limitation of logFC >0.5 and P value < 0.05.

### 2.12 Statistical analysis

Public databases were the major source of the involved data and no ethical consent was required. R 4.1.0 and R studio April 1, 1717 were utilized for the analyses mentioned above. Data visualization was achieved through R studio, GSEA 4.1.0. P-value<0.05 and q-value<0.05 were regarded as significant.

## 3 Results

### 3.1 Apoptosis and pyroptosis were the enriched and prognostic PCD in ccRCC

The mRNA expression matrix of ccRCC from TCGA together with the group information (539 tumor and 72 normal samples) were inputted into the GSEA software and delivered a result that necroptosis (q-value = 0.046), pyroptosis (q-value = 0.015) and apoptosis (q-value = 0.040) were significantly enriched in the ccRCC samples while the enrichment of autophagy and ferroptosis were not notable (Figs. S1A–E). The subsequent prognostic analysis demonstrated that these three enriched PCD could differentiate the overall survival of ccRCC (Figs. S1F–H). Thus, these three PCD scores were incorporated into multivariate cox regression analysis, inducing a model calculating PCD risk based on apoptosis and pyroptosis scores, PCD risk = −4.76 * (Apoptosis score) + 5.23 * (Pyroptosis score) (Fig. S1I).

### 3.2 High and low PCD risk groups present heterogeneous survival and immune features

**Fig. 2:**
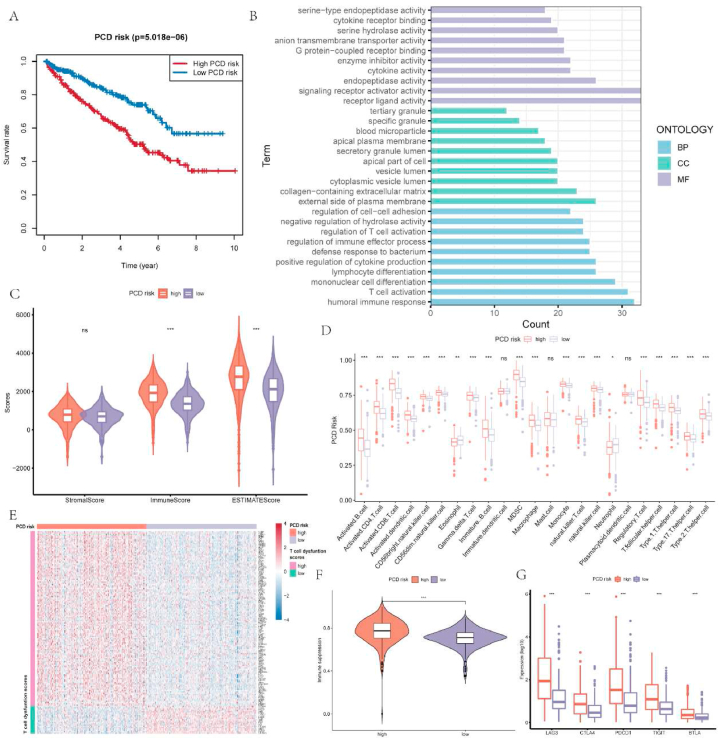
Different clinical and biological presentations of different PCD risk groups in ccRCC. A. Overall survival difference between different PCD risk groups. B. Results of functional enrichment analysis between PCD risk groups based on GO database. C. The differences of immune, stromal and ESTIMATE scores between PCD risk groups. D. Comparison of the infiltration levels of immune cells between PCD risk groups. E. Genes that were related to T-cell dysfunction scores in different PCD risk groups. F-G. The differences of immune suppression score and expression of LAG3, CTLA4, PDCD1, TIGIT, and BTLA between PCD risk groups.

Based on the mean of PCD risk, the ccRCC samples were separated into two groups with high and low PCD risk. According to Kaplan Meier survival analysis, samples with high PCD risk owned poorer prognosis than those with low PCD risk (Fig. 2A). Then, functional enrichment analysis was performed on these two groups. Various enriched biological processes were correlated to immunity in high PCD risk groups, including humoral immune response, regulation of cell-cell adhesion, regulation of immune effector process, and T cell activation. The enrichment of these processes may happen on cytoplasmic vesicle lumen, apical plasma membrane, and external side of the plasma membrane, covering some molecular functions like receptor ligand activity, endopeptidase activity, and cytokine activity (Fig. 2B). Given the high correlation between enriched function and immunity, we focused on the immune landscape of different PCD risk groups. The ESTIMATE results depicted that high immune scores and ESTIMATE scores were found in group with high PCD risk, indicating a high immune involvement and low tumor purity (Fig. 2C). CcRCC samples with high PCD risk had more infiltration of immune cells including both inflammatory immune cells and inhibitory immune cells (Fig. 2D). To further investigate the immune microenvironment in different PCD risk groups, we calculated the T-cell dysfunction scores and found that most genes with notably high T-cell dysfunction scores were differentially overexpressed in the high PCD risk group (Fig. 2E, Table S2). Meanwhile, in contrast to low PCD risk groups, the high PCD risk group exhibited heightened immune suppression scores as well as higher gene expression level of inhibitory immune checkpoint including LAG3, TIGIT, CTLA4, PDCD1, and BTLA (Fig. 2F–G). Therefore, the prognosis and suppressive immune microenvironment of ccRCC could be influenced by PCD risk.

### 3.3 Discovering PCD-related gene modules in ccRCC

After discovering that PCD was related to the prognosis and immune microenvironment of ccRCC, we intended to identify PCD-related genes that could cluster different PCD statuses to differentiate the prognosis of ccRCC. In order to discover PCD-related genes in ccRCC, differential expression analysis between ccRCC and normal samples was performed, obtaining 5136 DEGs. Then, a sample dendrogram was built up to identify the outliers, and those samples with a height greater than 6500 were excluded, leaving 486 samples for the following analysis (Fig. S2A). After that, the best soft threshold power was predicted as 3 based on the scale independence as well as mean connectivity (Figs. S2B–C). Based on this soft threshold power, the DEGs were clustered into multiple gene modules labeled by different colors (Fig. S2D), and these modules were correlated with pyroptosis and apoptosis scores respectively. It was revealed that the red and yellow modules had notably high correlations with both two prognostic PCD in ccRCC (Fig. S2E). Besides, as the module membership in these two modules became important, the genes became more significant for apoptosis (red, r = 0.85, p < 0.001; yellow, 0.67, p < 0.001) and pyroptosis (red, r = 0.88, p < 0.001; yellow, r = 0.77, p < 0.001; Figs. S2F–I). Therefore, we extracted the genes in the red and yellow modules as the PCD-related genes in ccRCC.

### 3.4 PCD-based molecular classification could differentiate ccRCC prognosis

**Fig. 3:**
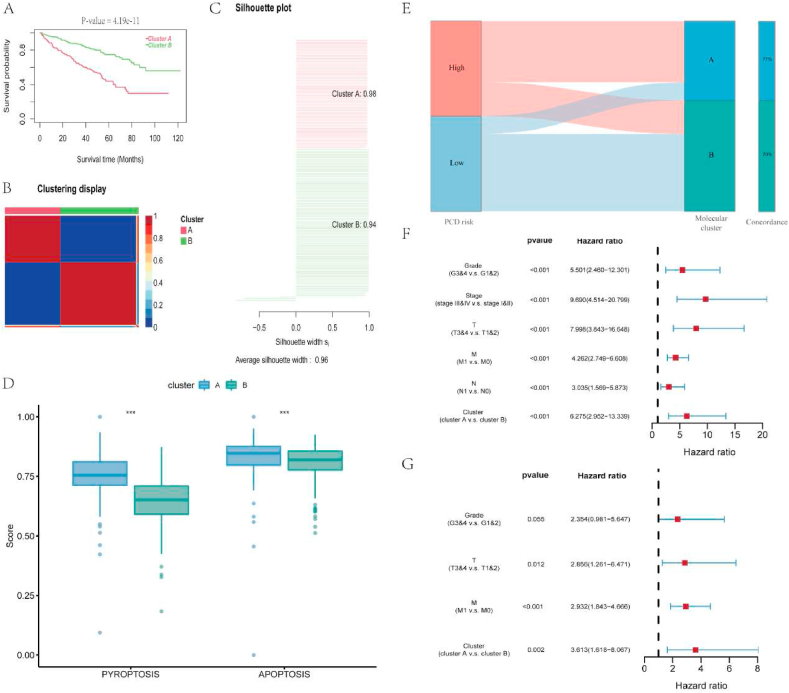
Identification of 2 PCD-related molecular clusters and their prognostic effect. A. Patients in cluster A had lower overall survival than those in cluster B. B–C. Cluster A was notably distinguished from cluster B and both of them had high silhouette scores. D. Differences of the pyroptosis and apoptosis scores between cluster A and B. E. The correlation between the PCD-related molecular clusters and PCD risk groups. F. The results of the univariate cox regression analysis. G. The results of multivariate cox regression analysis.

964 PCD-related genes were filtered through Kaplan Meier survival analysis, resulting in 292 genes related to the overall prognosis of ccRCC (Table S3). Then, these genes were used for CNMF classification and derived two molecular clusters of the ccRCC samples. Compared with cluster B, the overall survival of cluster A was notably lower (Fig. 3A). Besides, the silhouette scores of these two clusters (cluster A, 0.98; cluster B, 0.94; Fig. 3B–C) were high, meaning they were significantly distinct. When ccRCC samples were classified into 3 and 4 clusters, the silhouette scores for each cluster were relatively low. Thus, the classification of 2-cluster was selected (Figs. S3A–F). Comparing the PCD scores between these two clusters, we found that cluster A possessed higher pyroptosis and apoptosis scores than cluster B (Fig. 3D). Meanwhile, the molecular clusters were highly concordant with the PCD risk groups, indicating a good ability to distinguish PCD status (Fig. 3E). Besides, the chi-square test between the clusters illustrated that cluster A tended to have more male patients and patients with advanced grades and stages (Table 1). Thereafter, to discover the prognostic value of the PCD-based molecular classification, we included the clinical characteristics and the molecular clusters in univariate cox regression analysis followed by multivariate cox regression analysis. As the results shown, the PCD-based molecular classification could anticipate the prognosis of ccRCC independent of other factors and a worse prognosis would be happened in patients belong to high-PCD cluster (Fig. 3F–G).

**Table 1:** The difference in clinical features between cluster A and cluster B.

Clinical features		Cluster		P-value
		A	B	
Age	<65y	64	73	0.592
	≥65y	41	54	
Gender	Female	30	58	**0.008**
	Male	75	69	
Grade	G1&2	30	71	**<0.001**
	G3&4	75	56	
Stage	Stage I&II	36	88	**<0.001**
	Stage III&IV	69	39	
T	T1&2	45	91	**<0.001**
	T3&4	60	36	
N	N0	93	125	**0.002**
	N1	12	2	
M	M0	78	113	**0.004**
	M1	27	14	

### 3.5 High-PCD cluster is correlated with an abundant but suppressive immune microenvironment

**Fig. 4:**
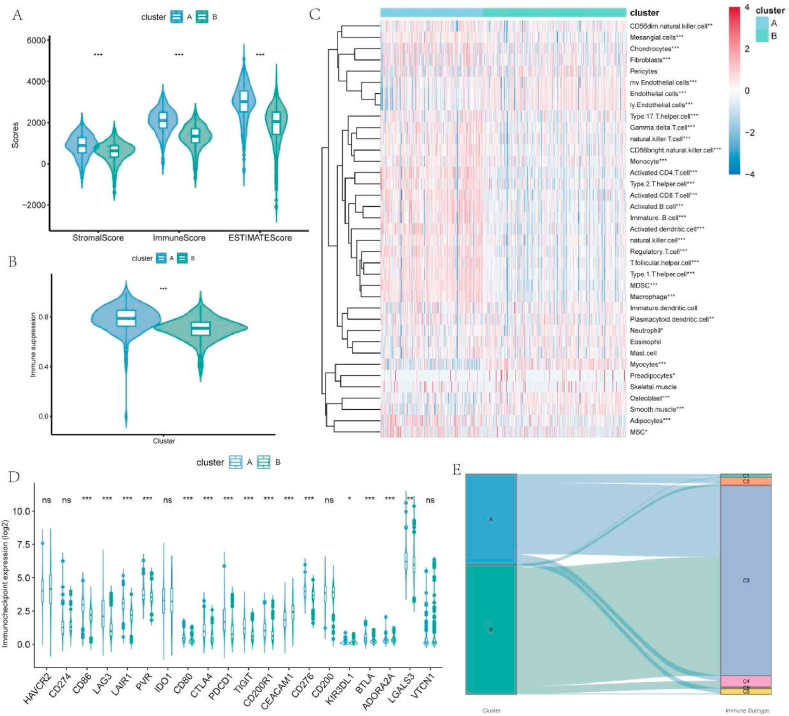
Differences in the immune landscape between molecular clusters. A. Different stromal score, immune score, and ESTIMATE score between cluster A and B. B. Cluster A had greater immune suppression scores than cluster B. C. Different molecular clusters exhibit different levels of infiltrated immune cells and stromal cells. D. Difference in expression of inhibitory immune checkpoints between molecular clusters. E. Correlation between the molecular clusters and previously identified immune subtypes.

Since the molecular clusters presented clinical diversity, we were interested in their difference in the TME of ccRCC. By comparing the results of ESTIMATE, it was revealed that cluster A exhibited a lower tumor purity than cluster B and both stromal and immune components were higher in cluster A (Fig. 4A). Analysis of the cellular infiltration demonstrated that some stromal cells (adipocyte, fibrocyte, and mesangial cell) tended to infiltrate in cluster A, while other stromal cells, like endothelial cell, lymphatic endothelial cell, and microvascular endothelial cell, were more infiltrated in cluster B. Meanwhile, although the inflammatory immune cells like activated CD8^+^ T cell, activated CD 4 T+ cell, activated B cell, and activated dendritic cell (DC), were highly infiltrated in cluster A, the inhibitory lymphocytes including regulatory T cell (Treg) and myeloid-derived suppressor cell (MDSC) were also more infiltrated in cluster A than cluster B (Fig. 4C). Concerning this considerable immune variation, we further investigated the immunity difference in these clusters. Higher immune suppression scores were identified in cluster A compared with cluster B (Fig. 4B), and multiple inhibitory immune inhibitor genes like PDCD1, CTLA4, TIGIT, LAG3, and CD80 were upregulated in cluster A (Fig. 4D). Meanwhile, most genes related to high T-cell dysfunction scores were significantly upregulated in cluster A (Fig. S3G). These may suggest a suppressive immune microenvironment in cluster A despite its abundant infiltrated immune cells. Moreover, we matched the current molecular classification with 6 immune subtypes discovered in the previous study [34]. Both clusters contain a substantial proportion of C3 (Fig. 4E), which was most common in kidney cancer and had a moderate level of tumor proliferation and elevated lymphocyte amount, reaching an immunologic control of cancer. However, cluster A possessed an unignorable scale of C2, C4, and C6. Although C2 was identified to be an immune-rich subtype, its tumor proliferation rate could surpass the immune response and was correlated with an unfavorable prognosis. Besides, C4 and C6 were the two subclasses with immunosuppressive tumor microenvironment and the worst outcome. Therefore, compared with cluster B, cluster A had a high level of PCD and may be correlated with an abundant but suppressive immune microenvironment.

### 3.6 High-PCD cluster is related to greater potency of generating immune response

**Fig. 5:**
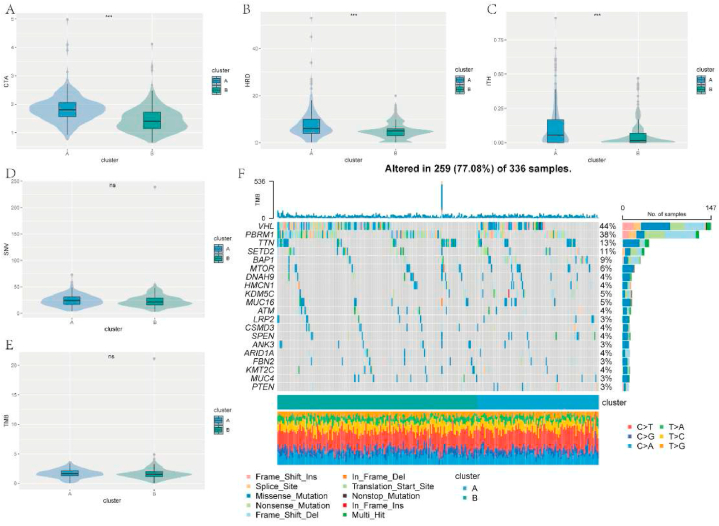
Analysis of the immunogenicity-related features in the molecular clusters. A-C. Different levels of CTA, HRD, and ITH among cluster A and B. D-E. No significant difference of SNV and TMB scores were recognized between the molecular clusters. F. A waterfall plot displaying the top 20 altered genes in cluster A and B.

For more understanding of the difference in the ability to generate immune response between the molecular clusters, several related features were introduced and analyzed. Compared with cluster B, higher values of CTA, HRD, and ITH were recognized in cluster A while the level of SNV and TMB was not significantly different (Fig. 5A–E). Meanwhile, the top 20 altered genes in these two clusters were identified and presented in the form of a waterfall chart (Fig. 5F). As the result showed, VHL (44%) and PBRM1 (38%) were the two most altered genes in both clusters followed by TTN (13%), SETD2 (11%), and BAP1 (9%). Moreover, the comparisons of immune chemokine, receptor, and MHC elucidated that the expression of these immunity-related factors was notably upregulated in cluster A (Fig. S3H). Thus, it may indicate that cluster A possessed a high potential to generate immune responses.

### 3.7 Patients in high-PCD cluster respond better toward immune and targeted therapies

**Fig. 6:**
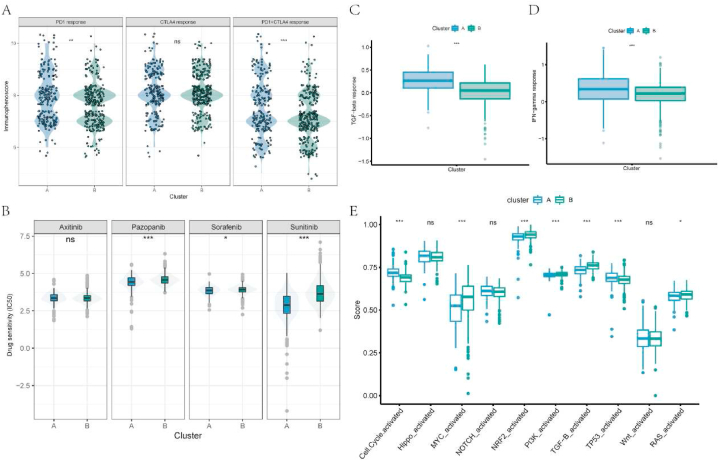
Investigation of the therapeutic response between molecular clusters. A. Differences of immunophenoscore of PD1, CTLA4 and PD1+CTLA4 blockades between the molecular clusters. B. Differences of IC50 of Axitinib, Pazopanib, Sorafenib, and Sunitinib among the molecular clusters. C-D. Patients in cluster A responded better to modulation of TGFβ and IFNγ signaling pathways than those in cluster B. E. Difference of 10 oncogenic pathways among cluster A and B.

Taking advantage of the online materials, we investigated the potential effect of the molecular clusters on the therapeutic decision. Through introducing immunophenoscore, which is positively correlated with therapeutic response to ICIs, it was demonstrated that samples in cluster A responded better toward PD1 blockade and combined blockade of PD1 and CTLA4 (Fig. 6A). Besides, the 50% inhibition concentration of Pazopanib, Sorafenib, and Sunitinib in cluster A was lower compared with those in cluster B, which means the samples in cluster A had higher sensitivity to these drugs (Fig. 6B). In addition, cluster A responded better to immune therapeutic measures like modulation of the TGF-beta pathway and IFN-gamma pathway (Fig. 6C–D). Furthermore, we investigated the enrichment of 10 oncogenic pathways in these clusters and identified that cell cycle and TP53 were more activated in cluster A (Fig. 6E), revealing a potential therapeutic target.

### 3.8 Validation of the molecular clusters

**Fig. 7:**
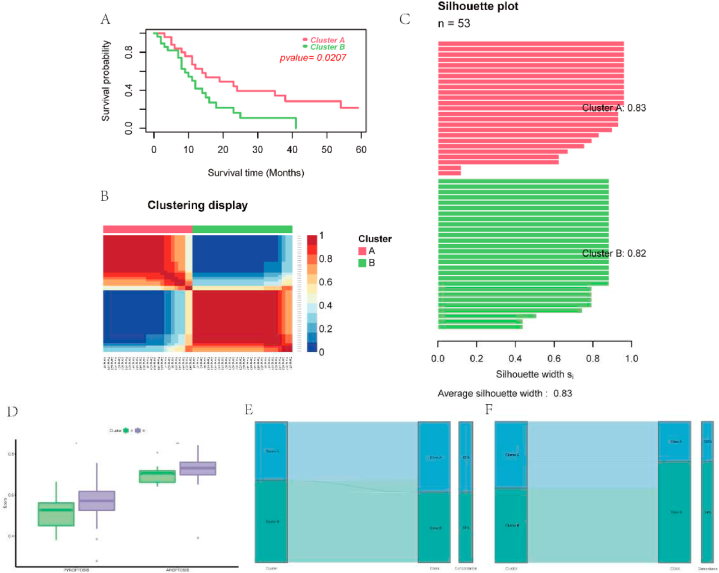
Validation of the molecular clusters and gene classifier. A. Patients in cluster A had higher overall survival than those in cluster B. B–C. Cluster A was notably distinguished from cluster B and both of them had high silhouette scores. D. The level of pyroptosis and apoptosis were higher in cluster A than those in cluster B. E-F. Correlation between the molecular clusters and gene classes in TCGA and E-MTAB-3267 cohorts respectively.

To support that the molecular clusters were reproducible, we extracted a separate ccRCC mRNA dataset, E-MTAB-3267, for validation. The prognostic PCD-related genes were utilized in the same way mentioned above to group the E-MTAB-3267 cohort into two molecular clusters whose overall survival was significantly different (Fig. 7A). Each cluster was independent and their silhouette scores were 0.83 and 0.82 respectively (Fig. 7B–C). Meanwhile, the classification of 3 and 4 clusters presented poor silhouette scores and were excluded (Figs. S4A–F). Besides, cluster B, whose overall survival was relatively low, had higher scores of pyroptosis and apoptosis than cluster A (Fig. 7D). These were all in line with the results of the molecular clusters constructed from the TCGA cohort. For the convenience of clinical usage, we made use of the NTP algorithm to produce a genetic classifier derived from the top 30 upregulated genes in each cluster (Table S4) and validated the classifier in TCGA and E-MTAB-3267 cohorts. The predicted results presented pleasant concordance with the molecular clusters (Fig. 7E–F) and had a similar outcome of survival analysis (Figs. S4G–H). demonstrating that the genetic classifier could conveniently and reliably represent the molecular classification. Furthermore, analysis of the transcriptome sequencing data from clinical samples indicated that most genes in the classifier were differentially expressed in ccRCC (Table 2), supporting the clinical application of the current gene classifier.

**Table 2:** Differential expression of the genes in the classifier between ccRCC and normal samples.

Gene	logFC	P value
AIM2	5.793991	0.000595
ADAMTS14	5.752162	1.49E-05
FCGR1A	5.059712	1.49E-05
CD72	4.823266	2.97E-05
BATF	4.72691	2.97E-05
GPR84	4.5812	0.001538
SLAMF8	4.450916	1.49E-05
TNFSF13B	4.100381	1.49E-05
CARD11	3.8562	1.49E-05
DOK3	3.654485	1.49E-05
SPI1	3.636505	1.49E-05
SH3BP1	3.596266	0.000178
JAK3	3.581513	1.49E-05
FOXP3	3.570916	5.94E-05
FMNL1	3.275528	1.49E-05
C1S	3.256804	1.49E-05
PYCARD	3.215839	2.97E-05
FCHO1	3.174602	0.000104
FERMT3	3.172103	1.49E-05
WAS	3.144529	2.97E-05
LY96	3.043022	1.49E-05
NCF4	2.925219	1.49E-05
DEF6	2.92065	2.97E-05
C1R	2.902083	0.000104
ADAM12	2.755361	0.001337
GMIP	2.714427	1.49E-05
RGS19	2.661285	1.49E-05
SERPINF1	2.221644	0.000178
RGS10	2.135263	0.000104
CDKL2	−0.828	0.018277
PRKD1	−1.23519	0.000951
CDKL1	−1.41258	0.000104
AUH	−1.57794	1.49E-05
BPHL	−1.64492	0.000951
NDRG2	−1.73313	1.49E-05
ANK3	−1.74848	0.000178
CLCN5	−1.84616	0.000654
L2HGDH	−1.90637	0.000446
FREM2	−1.91142	0.001506
C1orf210	−1.93118	0.000957
HIBCH	−1.96072	5.94E-05
PANK1	−2.06053	0.000282
PCCA	−2.2461	2.97E-05
EPB41L5	−2.29522	1.49E-05
CA4	−2.34603	0.000282
ACAT1	−2.4016	2.97E-05
WDR72	−2.4042	0.000104
NR3C2	−2.41463	1.49E-05
TRPM3	−2.43423	2.97E-05
MYL3	−2.79221	1.49E-05
ACADSB	−2.96291	1.49E-05
ALDH6A1	−3.86785	1.49E-05
TMEM174	−5.48751	0.000325

## 4 Discussion

As the major histological subtype of renal cancers, ccRCC would lead to an unfavorable prognosis at the advanced stage and poorly responds to traditional chemotherapy and radiotherapy [42]. Fortunately, novel immunotherapies such as ICIs were confirmed to be curative in ccRCC, though reliable biomarkers were required for predicting the therapeutic effect and toxicity [43]. ICIs exert their function by activating anti-tumor immunity to clear cancer cells [44]. With ongoing investigation, several PCDs, such as pyroptosis, were revealed to participate in the war against cancer and possessed potential synergistic effects on neoplastic therapies including immunotherapies [45]. In the current study, we investigate the status of programmed cell death in ccRCC and build up a cell death-based classifier to differentiate the survival and therapeutic response of ccRCC.

Through thorough analysis, apoptosis and pyroptosis were identified to be enriched in ccRCC and correlated with prognosis, among which apoptosis was positively related to survival and pyroptosis was associated with worse survival. Apoptosis has been widely investigated and demonstrated to be a biological process through which cells automatically step toward death under some particular stimuli. It is characterized by the condensation of chromatin and fragmentation of nuclear while the plasma membrane is intact during the whole process [46]. Involved in various diseases, apoptosis plays an important role in cell proliferation and survival and is critical for tumorigenesis. The evasion of apoptosis was one of the essential alterations for normal cells to transform into tumor cells [47]. Besides, apoptosis reduction, for example, by the downregulation of p53, was shown to be correlated with tumor growth and proliferation [48]. Nonetheless, as demonstrated in the results, cluster A with a high apoptosis level still had a worse prognosis than cluster B. This could partly due to the development of resistance to apoptosis in ccRCC. Mutation of the Von-Hippel Linda (VHL) gene, which happened in more than 70% of RCC patients, could protect RCC cells from apoptosis by preventing the degradation of hypoxia-inducible factor (HIF) [49]. Meanwhile, the NF-κB activation resulting from VHL mutation may induce an increased TNFα level which contributes to RIPK1-dependent inhibition of apoptosis in RCC cells [50].

Distinguished from apoptosis, pyroptosis is a kind of PCD that could induce inflammation. When undergoing pyroptosis, the cell membrane had pores formed on both sides and produced bubble-like protrusions leading to the rupture of the cell and release of pro-inflammatory molecules [51]. Pyroptosis recently had been found to be involved in carcinogenesis, and its role varies in different types of cancer [52]. Pyroptosis could be a protective factor for hepatocellular carcinoma, in which the expression of NLRP3, a pyroptosis-related protein, had a negative relationship with the clinical stage and pathologic grade [53]. Besides, AIM2 in HIV-infective cervical cancer cells could induce pyroptosis to protect against cancer cells [54]. However, as demonstrated by the above results, ccRCC patients and clusters with high pyroptosis as well as apoptosis levels were related to advanced clinical features and poor survival. There was a limited message about the relationship between pyroptosis and ccRCC, but the carcinogenic feature of pyroptosis in cancers had been identified. In breast cancer, the high expression of GSDMB, which is one of the pyroptosis effectors, was related to distance metastasis and poor prognosis [55]. Another effector, GSDMC, was found to be expressed in malignant melanoma and may participate in the invasive and metastatic features of the cancer cells [56]. Therefore, the enriched pyroptosis in ccRCC may serve as a pro-carcinogenic factor while the protective effect of apoptosis could be suppressed.

The carcinogenic effect of PCD could be achieved through modulating the tumor immune microenvironment [57] and our results also indicated the cluster with high levels of PCD was immune-rich with substantial infiltration of multiple types of immune cells. Although apoptosis is not inflammable, it induced the secretion of factors like ATP and lysophosphatidylcholine (LPC) to serve as chemo-attractants for the recruitment of immune cells to the neighboring microenvironment [58,59]. As an inflammable PCD, pyroptosis could induce the formation of multiple inflammasomes to modulate the immune system. Inflammasome NLRP3 was able to mediate IL-18 production and assist in the maturation of natural killer (NK) cells [60]. Besides, NLRP3 was essential for the production of IL-1β which could induce the priming of CD8^+^ T cells [61]. It was discovered that inducing pyroptosis was able to increase the infiltration of CD4^+^ T cells and CD8^+^ T cells [62]. However, despite the high level of inflammatory immune cells, the immune microenvironment in ccRCC with a high level of PCD was shown to be suppressive. It may be partially due to the co-infiltration of inhibitory immune cells in cluster A including MDSCs and Treg. Characterized by the co-expression of surface markers CD4, CD25, and FOXP3, Treg was revealed to be correlated with the poor prognosis of RCC probably through assisting the immune escape of tumor cells [63]. MDSC was another type of immune cell responsible for the suppressive state of anti-tumor T lymphocytes. Inflammasomes resulting from pyroptosis may facilitate the accumulation of these suppressive immune cells in TME [64]. Besides, Daley et al. found that pyroptosis could induce the differentiation of CD4^+^ T cells to inhibitory Th2 cells and Treg whereas the polarization of Th1 cells was suppressed [65]. Moreover, the overexpression of MHC-I molecules in the high cell death cluster could attribute to the suppressive environment. RCC cells expressing HLA-G possessed higher resistance toward the cytotoxic effect of CD8^+^ T cells and NK [66]. Meanwhile, HLA-E may serve as a promoter for RCC to escape from immune surveillance [67]. The inhibitory immune checkpoint is an additional mechanism through which tumor cells achieve immune evasion. PD-1 expression on lymphocytes could interact with the ligand of PD-1 on tumor cells to conduct suppressive signals to the anti-tumor lymphocytes and protect the tumor cells from the immune response. CTLA-4 is another popular inhibitory immune checkpoint that could competitively bind CD80 and CD86 against CD28, interrupting the activation of anti-tumor immunity [68]. The unregulated expression of these two inhibitory checkpoints together with others such as LAG3 and TIM3 may further support the suppressive immune microenvironment in ccRCC with a high level of PCD. Thus, it seems that the counterbalance between inflammatory and inhibitory immune cells in ccRCC with high PCD levels was inclined to the suppressive one.

Despite the suppressive immune microenvironment, ccRCC with high cell death levels respond better to ICIs thanks to the considerable immune reservoir and immunogenicity. Considering ICIs function through reactivating the anti-tumor immune system, a TME with substantial infiltrating cytotoxic immune cells was preferred for ICIs therapy [69]. In addition, the ability to generate tumor-associated antigens was also essential for ICIs to exert functions. Cancer testis antigen (CTA) was a kind of tumor-associated antigen specifically expressed in tumor tissues. A notable relationship had been identified between CTA and the activation of cytotoxic T lymphocytes as well as their anti-tumor ability, representing the immunogenicity of CTA [70]. Homologous recombination is important for DNA to stay intact and it was frequently disrupted in cancers. Homologous recombination deficiency (HRD) enhanced the potential of tumors to generate neoantigens, which could enhance the efficacy of ICIs [15]. Although high intratumoral heterogeneity (ITH) potentiates the tumor cells to proliferate and progress, it could be a reliable target for developing combination therapies of ICIs with greater efficacy [71]. With this concern, the high PCD cluster could not only instruct the monotherapy of ICIs in ccRCC but also could facilitate the combination therapies with other curative drugs. Tyrosine kinase inhibitors (TKIs) also belong to the FDA-approved therapies for ccRCC. In addition, to act as one of the first-line therapies for ccRCC, TKIs were demonstrated to reprogram the immune microenvironment and synergize with ICIs [72]. Distinguished from pre-treatment examinations, the amount of Treg decreased while the level of IFNγ-producing CD4^+^ lymphocytes increased after 2 cycles of Sunitinib administration [73]. Meanwhile, the proportion of MDSCs in ccRCC could also be negatively modulated after TKIs therapy [74]. These may serve as a biological basis to support the combined administration of TKIs and ICIs. A recent study discovered that ICIs therapy achieved better efficacy in patients pre-treated with TKI [75]. Except for this kind of combination, the current study also provided some potential therapeutic choices. TGF-β was revealed to act as a suppressor for adaptive immune response by inducing Treg proliferation and interrupting the function of cytotoxic lymphocytes [76]. On the contrary, IFN-γ could assist the recognition of tumor cells by the immune system to enhance anti-tumor function [77]. Considering that patients with a high level of PCD respond well to the modulation of TGF-beta and IFN-gamma, it would be beneficial to combine them with ICIs therapy. Moreover, cell cycle-related pathway and p53 signaling were enriched in the high PCD group. The signaling of p53 was essential for the cell cycle, apoptosis, and drug resistance. The modulation of p53 could promote the sorafenib response in RCC cells and induce cell apoptosis [78], providing additional selection for combination therapies in ccRCC.

After all, the current study thoroughly investigated the PCD status in ccRCC and uncover the immune-modulating and prognostic roles of apoptosis and pyroptosis. A simple and reproducible PCD-based gene classifier was constructed to help clinicians differentiate the prognosis of ccRCC patients and prescript individualized therapeutic strategies, including deciding which drug could be used and how these drugs can be used in combination. At the same time, our results could support further research on the mechanism and functions of PCD in ccRCC and the discovery of novel therapeutic targets. Still, there are limitations existing in the current study. Most of the analyses were based on public datasets, TCGA and E-MTAB-3267 because of their intact prognosis data. Although we have validated the gene classifier with transcriptomic data from clinical samples, a larger clinical cohort with survival and treatment information would be beneficial to confirm the prognostic and therapeutic predictive ability of this classifier. Meanwhile, despite we have proposed some potential functional mechanisms of PCD and its relationship with the TME of ccRCC, profound cell and functional experiments are necessary to clarify these mechanisms and connections. Moreover, based on further validation and experiments, the current gene classifier can progress toward more concise and convenient clinical application.

## 5 Conclusion

In the current study, we comprehensively investigated the status of PCD in ccRCC and constructed a PCD-based classification with distinct clinical features and TME heterogenicity. It could facilitate the clinical diagnosis and individualized treatment of ccRCC, shedding light on further research on combination therapies.

## Author contribution statement

Yanlin Tang, Changzheng Zhang: Conceived and designed the experiments; Analyzed and interpreted the data; Wrote the paper.

Chujin Ye, Kaiwen Tian: Performed the experiments; Wrote the paper.

Jiayi Zeng, Shouyu Cheng, Weinan Zeng, Bowen Yang: Contributed reagents, materials, analysis tools or data; Wrote the paper.

Yanjun Liu, Yuming Yu: Performed the experiments; Contributed reagents, materials, analysis tools or data; Wrote the paper.

## Data availability statement

The data and information demonstrated and analyzed throughout the present study were obtained from the Genomics Data Commons Data Portal (https://portal.gdc.cancer.gov/), Gene Set Enrichment Analysis (http://www.gsea-msigdb.org/gsea/index.jsp), xCell (https://xcell.ucsf.edu/), the cBioPortal for Cancer Genomics (https://www.cbioportal.org/), The Cancer Immunome Database (https://tcia.at/home), Genomics of Drug Sensitivity in Cancer (https://www.cancerrxgene.org), ArrayExpress (https://www.ebi.ac.uk/arrayexpress/). The patient information of the clinical transcriptome data could be accessed from the supplementary material (Table S1).

## Ethical statement

The authors are accountable for all aspects of the work in ensuring that questions related to the accuracy or integrity of any part of the work are appropriately investigated and resolved. The studies involving human participants were reviewed and approved by Ethics Committee of Guangdong Provincial People's Hospital. The patients provided their written informed consent to participate in this study.

## Declaration of competing interest

The authors declare that they have no known competing financial interests or personal relationships that could have appeared to influence the work reported in this paper.

## Abbreviation

**ccRCC**: Clear cell renal cell carcinoma
**ICIs**: immune checkpoint inhibitors
**PCD**: programmed cell death
**TME**: tumor microenvironment
**TCGA**: The Cancer Genome Atlas
**CTA**: cancer testis antigens
**HRD**: homologous recombination repair deficiency
**ITH**: intratumoral heterogeneity
**SNV**: single-nucleotide variation
**TIDE**: tumor immune dysfunction and evasion
**TMB**: tumor mutation burden
**MDSC**: myeloid-derived suppressor cell
**Treg**: regulatory T cell
**WGCNA**: Weight gene correlation network analysis
**CNMF**: non-negative matrix factorization
**ESTIMATE**: Estimation of Stromal and Immune cells in Malignant Tumor tissues using Expression data
**ssGSEA**: single-sample Gene Set Enrichment Analysis
