# Supplementary material for: Construction and validation of programmed cell death-based molecular clusters for prognostic and therapeutic significance of clear cell renal cell carcinoma
Source: Heliyon. 2023 May 2;9(5):e15693. doi: 10.1016/j.heliyon.2023.e15693 (PMC10256830; doi:10.1016/j.heliyon.2023.e15693)
Supplement: Multimedia component 1 [file mmc1.zip › Table S2.docx]

| Table S2. Genes with notable T-cell dysfunction scores in PCD risk groups. | | | |
| --- | --- | --- | --- |
| **Gene** | **T-cell dysfunction score** | **p-value** | **FDR** |
| KCNN4 | 7.343808 | 2.08E-13 | 8.72E-11 |
| CLMP | 7.11857 | 1.09E-12 | 2.29E-10 |
| SLAMF9 | 6.744715 | 1.53E-11 | 2.15E-09 |
| MYBL2 | 6.60382 | 4.01E-11 | 4.21E-09 |
| GPR84 | 6.34013 | 2.30E-10 | 1.76E-08 |
| AIM2 | 6.314795 | 2.71E-10 | 1.76E-08 |
| ZIC2 | 6.263921 | 3.75E-10 | 1.97E-08 |
| RGS20 | 6.170403 | 6.81E-10 | 3.18E-08 |
| PTTG1 | 6.128475 | 8.87E-10 | 3.73E-08 |
| IGF2BP2 | 6.025206 | 1.69E-09 | 5.92E-08 |
| RNASE2 | 6.024864 | 1.69E-09 | 5.92E-08 |
| IGF2BP3 | 5.952162 | 2.65E-09 | 8.55E-08 |
| MELTF | 5.835593 | 5.36E-09 | 1.61E-07 |
| C1R | 5.771487 | 7.86E-09 | 2.20E-07 |
| UBE2C | 5.734001 | 9.81E-09 | 2.57E-07 |
| HAMP | 5.672657 | 1.41E-08 | 3.47E-07 |
| MUC12 | 5.63539 | 1.75E-08 | 4.05E-07 |
| CXCL5 | 5.627087 | 1.83E-08 | 4.05E-07 |
| SLC17A9 | 5.345446 | 9.02E-08 | 1.89E-06 |
| IFNG | 5.321751 | 1.03E-07 | 2.06E-06 |
| SNORC | 5.280654 | 1.29E-07 | 2.46E-06 |
| C1S | 5.189171 | 2.11E-07 | 3.86E-06 |
| IGFLR1 | 5.085247 | 3.67E-07 | 6.43E-06 |
| ZIC5 | 4.981528 | 6.31E-07 | 1.06E-05 |
| TRNP1 | 4.946533 | 7.55E-07 | 1.21E-05 |
| CPNE7 | 4.901966 | 9.49E-07 | 1.42E-05 |
| C6orf141 | 4.789866 | 1.67E-06 | 2.35E-05 |
| WFDC10B | 4.78831 | 1.68E-06 | 2.35E-05 |
| FDCSP | 4.773343 | 1.81E-06 | 2.45E-05 |
| TUBB3 | 4.760584 | 1.93E-06 | 2.53E-05 |
| CASP5 | 4.676979 | 2.91E-06 | 3.71E-05 |
| UCHL1 | 4.62548 | 3.74E-06 | 4.62E-05 |
| CCNO | 4.544054 | 5.52E-06 | 6.62E-05 |
| LAG3 | 4.528775 | 5.93E-06 | 6.92E-05 |
| PRAME | 4.49835 | 6.85E-06 | 7.77E-05 |
| ANKRD33 | 4.478012 | 7.53E-06 | 8.21E-05 |
| INHBE | 4.469382 | 7.84E-06 | 8.24E-05 |
| RORB | 4.356312 | 1.32E-05 | 0.000135 |
| TNFSF14 | 4.352215 | 1.35E-05 | 0.000135 |
| MAGEA10 | 4.315647 | 1.59E-05 | 0.000155 |
| ANGPTL8 | 4.279131 | 1.88E-05 | 0.000179 |
| PLXNB3 | 4.262159 | 2.02E-05 | 0.000189 |
| BATF | 4.195621 | 2.72E-05 | 0.000248 |
| CLVS1 | 4.166953 | 3.09E-05 | 0.000276 |
| BTBD11 | 4.13519 | 3.55E-05 | 0.00031 |
| TREM1 | 4.119675 | 3.79E-05 | 0.000325 |
| CXCL13 | 4.096274 | 4.20E-05 | 0.000346 |
| CCN5 | 4.076437 | 4.57E-05 | 0.00035 |
| KLF17 | 4.07533 | 4.59E-05 | 0.00035 |
| SAGE1 | 4.072495 | 4.65E-05 | 0.00035 |
| COL4A6 | 4.071673 | 4.67E-05 | 0.00035 |
| PYCARD | 4.071411 | 4.67E-05 | 0.00035 |
| MNX1 | 4.064841 | 4.81E-05 | 0.000354 |
| RUFY4 | 4.048186 | 5.16E-05 | 0.000374 |
| FCGR1A | 3.93127 | 8.45E-05 | 0.000597 |
| IGFL2 | 3.902093 | 9.54E-05 | 0.000653 |
| CHI3L2 | 3.898234 | 9.69E-05 | 0.000653 |
| C1QL1 | 3.895716 | 9.79E-05 | 0.000653 |
| RTP5 | 3.869704 | 0.000109 | 0.000715 |
| ADAM8 | 3.785773 | 0.000153 | 0.000975 |
| APOL1 | 3.757022 | 0.000172 | 0.001078 |
| IL27 | 3.738608 | 0.000185 | 0.001143 |
| IL20RB | 3.727642 | 0.000193 | 0.001176 |
| HCST | 3.714743 | 0.000203 | 0.00122 |
| ITIH4 | 3.709423 | 0.000208 | 0.001229 |
| C6orf118 | 3.705403 | 0.000211 | 0.001231 |
| TNFRSF18 | 3.665238 | 0.000247 | 0.001422 |
| SMIM23 | 3.592214 | 0.000328 | 0.001861 |
| ZDHHC22 | 3.585733 | 0.000336 | 0.001882 |
| CCL5 | 3.576214 | 0.000349 | 0.001927 |
| PPP1R1A | 3.522913 | 0.000427 | 0.002298 |
| SLAMF8 | 3.511408 | 0.000446 | 0.00237 |
| PITX1 | 3.507941 | 0.000452 | 0.002371 |
| ACHE | 3.486767 | 0.000489 | 0.002504 |
| CRP | 3.452181 | 0.000556 | 0.002786 |
| PTPN7 | 3.451613 | 0.000557 | 0.002786 |
| CD7 | 3.440207 | 0.000581 | 0.002872 |
| BCL2A1 | 3.367291 | 0.000759 | 0.003665 |
| AQP9 | 3.35454 | 0.000795 | 0.003794 |
| ITPKA | 3.348656 | 0.000812 | 0.003832 |
| MCEMP1 | 3.34334 | 0.000828 | 0.003863 |
| KCNH3 | 3.331128 | 0.000865 | 0.003992 |
| IGLL5 | 3.29919 | 0.00097 | 0.004427 |
| CLIC3 | 3.286218 | 0.001015 | 0.004586 |
| ERC2 | 3.248191 | 0.001161 | 0.005189 |
| NEUROG3 | 3.204853 | 0.001351 | 0.005958 |
| ZP1 | 3.202623 | 0.001362 | 0.005958 |
| ATP8B3 | 3.176481 | 0.001491 | 0.006455 |
| XCL1 | 3.157122 | 0.001593 | 0.00676 |
| FRMPD3 | 3.100847 | 0.00193 | 0.008024 |
| MARCO | 3.094589 | 0.001971 | 0.008115 |
| ZBP1 | 3.083387 | 0.002047 | 0.008186 |
| HOXC12 | 3.049934 | 0.002289 | 0.009069 |
| PLA2G4D | 3.005791 | 0.002649 | 0.010398 |
| SAA4 | 2.991009 | 0.002781 | 0.010813 |
| JSRP1 | 2.960796 | 0.003068 | 0.01179 |
| MTTP | 2.958841 | 0.003088 | 0.01179 |
| ANXA8 | 2.952066 | 0.003157 | 0.011944 |
| GBP5 | 2.916558 | 0.003539 | 0.013272 |
| CIDEC | 2.892628 | 0.00382 | 0.014199 |
| PTPRH | 2.870298 | 0.004101 | 0.015108 |
| CPN2 | 2.8638 | 0.004186 | 0.015171 |
| TTC24 | 2.863479 | 0.00419 | 0.015171 |
| PLA2G2C | 2.848698 | 0.00439 | 0.015758 |
| TM4SF19 | 2.819173 | 0.004815 | 0.017137 |
| CPA4 | 2.810325 | 0.004949 | 0.017468 |
| GOLGA6L2 | 2.78647 | 0.005329 | 0.018496 |
| SLN | 2.78269 | 0.005391 | 0.018559 |
| TIGIT | 2.704648 | 0.006838 | 0.023348 |
| SIRPG | 2.689671 | 0.007152 | 0.024225 |
| XCL2 | 2.630737 | 0.00852 | 0.0284 |
| UGT1A10 | 2.601812 | 0.009273 | 0.030667 |
| SH2D2A | 2.577673 | 0.009947 | 0.032638 |
| LTA | 2.518053 | 0.011801 | 0.03842 |
| CNPY1 | 2.473771 | 0.01337 | 0.043171 |
| SAA2-SAA4 | 2.471222 | 0.013465 | 0.043171 |
| SPIB | 2.447369 | 0.01439 | 0.045787 |
| FOXP3 | 2.425875 | 0.015272 | 0.048226 |
| NIPAL4 | 2.409129 | 0.015991 | 0.049749 |
| CALHM6 | 2.395702 | 0.016589 | 0.051229 |
| GOLGA6L7 | 2.385688 | 0.017047 | 0.052262 |
| NBL1 | 2.377043 | 0.017452 | 0.053115 |
| ATP2B3 | 2.330036 | 0.019804 | 0.05984 |
| LRG1 | 2.262231 | 0.023683 | 0.071049 |
| IL2RG | 2.229157 | 0.025803 | 0.07632 |
| ZNF683 | 2.213062 | 0.026893 | 0.078837 |
| PRKCG | 2.211087 | 0.02703 | 0.078837 |
| MYBPH | 2.181897 | 0.029117 | 0.084099 |
| MMP8 | 2.177616 | 0.029435 | 0.084099 |
| KLK2 | 2.14863 | 0.031664 | 0.088658 |
| RNASE3 | 2.120044 | 0.034002 | 0.094576 |
| TNNT1 | 2.09819 | 0.035888 | 0.098517 |
| ZNF804B | -2.11187 | 0.034697 | 0.095874 |
| FRAS1 | -2.15937 | 0.030822 | 0.08688 |
| ZNF728 | -2.16058 | 0.030728 | 0.08688 |
| FSTL4 | -2.17794 | 0.02941 | 0.084099 |
| BMP5 | -2.23022 | 0.025733 | 0.07632 |
| SCN4A | -2.42107 | 0.015475 | 0.048503 |
| ERVW-1 | -2.66371 | 0.007728 | 0.025968 |
| PIEZO2 | -2.79673 | 0.005162 | 0.018068 |
| SHISA6 | -3.08599 | 0.002029 | 0.008186 |
| SCD5 | -3.09086 | 0.001996 | 0.008138 |
| DGKI | -3.15314 | 0.001615 | 0.006784 |
| USP2 | -3.16024 | 0.001576 | 0.006756 |
| TOX3 | -3.37068 | 0.00075 | 0.003662 |
| PLG | -3.49837 | 0.000468 | 0.002427 |
| CA4 | -3.57233 | 0.000354 | 0.00193 |
| SLC10A6 | -3.80989 | 0.000139 | 0.000898 |
| SLC6A19 | -3.9292 | 8.52E-05 | 0.000597 |
| F2RL3 | -4.09739 | 4.18E-05 | 0.000346 |
| APOLD1 | -4.47551 | 7.62E-06 | 8.21E-05 |
| ATP11A | -4.94161 | 7.75E-07 | 1.21E-05 |
| SLC16A12 | -6.3024 | 2.93E-10 | 1.76E-08 |
