# Supplementary material for: Construction and validation of programmed cell death-based molecular clusters for prognostic and therapeutic significance of clear cell renal cell carcinoma
Source: Heliyon. 2023 May 2;9(5):e15693. doi: 10.1016/j.heliyon.2023.e15693 (PMC10256830; doi:10.1016/j.heliyon.2023.e15693)
Supplement: Multimedia component 1 [file mmc1.zip › Table S3.docx]

Table S3. Prognostic PCD-related genes in ccRCC.

| gene | KM | Pvalue |
| --- | --- | --- |
| FCER1G | 0.008743 | <0.05 |
| C1QA | 0.007848 | <0.05 |
| FCGR1A | 0.002372 | <0.05 |
| C1QB | 0.016511 | <0.05 |
| LAIR1 | 0.020117 | <0.05 |
| LILRB2 | 0.011661 | <0.05 |
| GPR84 | 1.96E-05 | <0.05 |
| TRPM2 | 0.003163 | <0.05 |
| TNFSF13B | 0.000181 | <0.05 |
| NCF4 | 0.000523 | <0.05 |
| OSCAR | 0.00041 | <0.05 |
| GNA15 | 0.004414 | <0.05 |
| LILRB1 | 0.04706 | <0.05 |
| EVI2A | 0.023219 | <0.05 |
| CTSZ | 0.002104 | <0.05 |
| TREM2 | 0.010161 | <0.05 |
| CASP5 | 0.007701 | <0.05 |
| LY96 | 0.004808 | <0.05 |
| ADAP2 | 0.00274 | <0.05 |
| HK3 | 0.002487 | <0.05 |
| RNASE2 | 3.47E-05 | <0.05 |
| PLB1 | 0.003747 | <0.05 |
| IFI16 | 9.32E-05 | <0.05 |
| C1R | 3.79E-09 | <0.05 |
| C1S | 2.86E-07 | <0.05 |
| LHFPL2 | 0.026908 | <0.05 |
| FCGR2B | 0.002816 | <0.05 |
| CHST11 | 0.011015 | <0.05 |
| CD80 | 0.012484 | <0.05 |
| IFNGR2 | 0.004408 | <0.05 |
| OSM | 0.001572 | <0.05 |
| EMILIN2 | 0.044692 | <0.05 |
| TOM1L2 | 0.00052 | <0.05 |
| ATP2B2 | 0.012289 | <0.05 |
| SLC9A9 | 0.01276 | <0.05 |
| RCSD1 | 0.026415 | <0.05 |
| THEMIS2 | 0.028904 | <0.05 |
| SIRPD | 0.017335 | <0.05 |
| APOC1 | 0.01814 | <0.05 |
| AQP9 | 0.000123 | <0.05 |
| GBGT1 | 0.008697 | <0.05 |
| ALOX5 | 0.005817 | <0.05 |
| HAMP | 5.47E-06 | <0.05 |
| PLEKHG3 | 0.006872 | <0.05 |
| TGM2 | 0.006776 | <0.05 |
| C3 | 0.03655 | <0.05 |
| USP2 | 0.013921 | <0.05 |
| LIPA | 0.000137 | <0.05 |
| MARCO | 0.021521 | <0.05 |
| GLIPR1 | 0.00343 | <0.05 |
| SLC11A1 | 7.05E-06 | <0.05 |
| CEACAM4 | 0.005589 | <0.05 |
| SMIM24 | 0.000111 | <0.05 |
| LILRA4 | 4.34E-05 | <0.05 |
| RNASE3 | 0.014567 | <0.05 |
| CLEC2B | 2.77E-05 | <0.05 |
| CHI3L2 | 5.11E-06 | <0.05 |
| PTH1R | 0.000636 | <0.05 |
| PEBP1 | 0.026707 | <0.05 |
| SLFN11 | 0.000299 | <0.05 |
| NRXN2 | 0.010352 | <0.05 |
| TM6SF1 | 0.022702 | <0.05 |
| PREX1 | 0.003294 | <0.05 |
| METTL27 | 0.012147 | <0.05 |
| SLC22A13 | 0.000104 | <0.05 |
| ENTPD2 | 0.003176 | <0.05 |
| TGFBI | 0.004317 | <0.05 |
| SLC6A19 | 2.29E-06 | <0.05 |
| RPS20 | 0.000455 | <0.05 |
| RNF149 | 0.007725 | <0.05 |
| ADAMTSL4 | 0.00036 | <0.05 |
| SLC22A6 | 6.37E-07 | <0.05 |
| CLEC18A | 6.04E-05 | <0.05 |
| IBSP | 2.83E-05 | <0.05 |
| CYP4A22 | 0.000226 | <0.05 |
| SLC22A12 | 0.000115 | <0.05 |
| CRYM | 0.011363 | <0.05 |
| SAA2-SAA4 | 6.16E-07 | <0.05 |
| LGALS12 | 0.015228 | <0.05 |
| AKR7A3 | 9.03E-05 | <0.05 |
| FDCSP | 0.000315 | <0.05 |
| ENKUR | 0.009941 | <0.05 |
| ADH6 | 0.001168 | <0.05 |
| PCK1 | 2.40E-05 | <0.05 |
| SAA4 | 2.69E-07 | <0.05 |
| CLEC18C | 0.001276 | <0.05 |
| TMEM174 | 0.000219 | <0.05 |
| PAQR7 | 0.009539 | <0.05 |
| VNN3 | 0.001809 | <0.05 |
| ADGRG3 | 0.002583 | <0.05 |
| TMEM82 | 4.00E-06 | <0.05 |
| DHDH | 3.00E-05 | <0.05 |
| PF4V1 | 0.032045 | <0.05 |
| SLC8A1 | 0.023104 | <0.05 |
| CMTM2 | 0.035945 | <0.05 |
| CYP1B1 | 0.013405 | <0.05 |
| ANGPTL3 | 0.009677 | <0.05 |
| KCNK10 | 0.012781 | <0.05 |
| DOCK4 | 0.004512 | <0.05 |
| TLR3 | 9.20E-07 | <0.05 |
| MGARP | 0.010574 | <0.05 |
| NEK6 | 0.003299 | <0.05 |
| LIPI | 8.54E-05 | <0.05 |
| RGS19 | 0.00034 | <0.05 |
| RAC2 | 0.009666 | <0.05 |
| SPI1 | 0.003743 | <0.05 |
| FERMT3 | 0.018605 | <0.05 |
| TNFAIP8L2 | 0.002458 | <0.05 |
| GPSM3 | 0.010388 | <0.05 |
| CORO1A | 0.010183 | <0.05 |
| WAS | 0.014071 | <0.05 |
| CYTH4 | 0.002377 | <0.05 |
| LGALS9 | 0.025877 | <0.05 |
| CASP4 | 0.001236 | <0.05 |
| SH3BP1 | 0.027619 | <0.05 |
| FCGR1B | 0.000278 | <0.05 |
| LST1 | 0.017528 | <0.05 |
| PPP1R18 | 1.64E-05 | <0.05 |
| RGS10 | 5.23E-05 | <0.05 |
| BATF | 8.85E-05 | <0.05 |
| FMNL1 | 0.001779 | <0.05 |
| HCST | 7.57E-06 | <0.05 |
| TIGIT | 0.041855 | <0.05 |
| SLA2 | 0.039122 | <0.05 |
| SP140 | 0.00859 | <0.05 |
| CCL5 | 0.010358 | <0.05 |
| PLEKHO1 | 4.26E-05 | <0.05 |
| EFHD2 | 9.85E-05 | <0.05 |
| DOK3 | 0.000144 | <0.05 |
| SLAMF1 | 0.003675 | <0.05 |
| CSF1 | 0.002814 | <0.05 |
| RELT | 0.001786 | <0.05 |
| PARVG | 0.016196 | <0.05 |
| DOK1 | 0.000953 | <0.05 |
| CD72 | 0.002289 | <0.05 |
| DEF6 | 0.000272 | <0.05 |
| STAC3 | 1.64E-05 | <0.05 |
| NOD2 | 0.000234 | <0.05 |
| PSMB10 | 0.009726 | <0.05 |
| GBP2 | 0.019746 | <0.05 |
| EBI3 | 0.009614 | <0.05 |
| PCED1B | 0.01488 | <0.05 |
| PTPN7 | 0.011959 | <0.05 |
| CKLF | 0.000348 | <0.05 |
| SOWAHD | 0.002746 | <0.05 |
| JAK3 | 6.04E-09 | <0.05 |
| LTB | 0.00426 | <0.05 |
| LIMD2 | 0.000466 | <0.05 |
| ARRB2 | 0.020201 | <0.05 |
| SH2D2A | 5.00E-05 | <0.05 |
| ARHGAP9 | 0.00202 | <0.05 |
| FOXP3 | 0.000183 | <0.05 |
| XCL2 | 0.031634 | <0.05 |
| LAG3 | 0.003049 | <0.05 |
| IL15RA | 0.000361 | <0.05 |
| MCUB | 0.015171 | <0.05 |
| TBC1D10C | 0.017349 | <0.05 |
| IL27 | 0.003202 | <0.05 |
| BID | 0.001075 | <0.05 |
| TMC8 | 0.002411 | <0.05 |
| PYCARD | 9.74E-05 | <0.05 |
| CCM2 | 5.30E-06 | <0.05 |
| CXCL13 | 0.000903 | <0.05 |
| IFNG | 0.006365 | <0.05 |
| HAPLN3 | 0.002119 | <0.05 |
| SIPA1 | 0.003016 | <0.05 |
| REEP4 | 7.45E-06 | <0.05 |
| L2HGDH | 0.000213 | <0.05 |
| LILRB3 | 0.001172 | <0.05 |
| XCL1 | 0.005106 | <0.05 |
| CTLA4 | 0.006222 | <0.05 |
| EPB41L5 | 4.27E-06 | <0.05 |
| TYMP | 0.005155 | <0.05 |
| MAP4K1 | 0.048678 | <0.05 |
| CDKL2 | 5.12E-06 | <0.05 |
| ACADSB | 2.55E-07 | <0.05 |
| ALDH6A1 | 4.57E-09 | <0.05 |
| PLCB2 | 0.003203 | <0.05 |
| FXYD5 | 0.000165 | <0.05 |
| TNFRSF9 | 0.049329 | <0.05 |
| CCDC88B | 0.000196 | <0.05 |
| APOBEC3H | 0.003031 | <0.05 |
| RHBDF2 | 0.01908 | <0.05 |
| NFE2L3 | 3.50E-06 | <0.05 |
| CARD9 | 9.94E-05 | <0.05 |
| OASL | 0.001215 | <0.05 |
| ISG20 | 0.003801 | <0.05 |
| C2 | 0.033451 | <0.05 |
| IGFLR1 | 5.83E-08 | <0.05 |
| ZBP1 | 0.001705 | <0.05 |
| ADAM8 | 2.73E-06 | <0.05 |
| SEPTIN1 | 0.018251 | <0.05 |
| TNFRSF18 | 1.70E-06 | <0.05 |
| STAT4 | 0.014129 | <0.05 |
| CNNM2 | 1.19E-06 | <0.05 |
| APOC2 | 0.00037 | <0.05 |
| DTX2 | 9.28E-06 | <0.05 |
| FRK | 0.000163 | <0.05 |
| JAKMIP1 | 0.037124 | <0.05 |
| GPR25 | 0.016826 | <0.05 |
| WLS | 8.39E-06 | <0.05 |
| ARHGAP22 | 1.16E-05 | <0.05 |
| HIBCH | 1.29E-06 | <0.05 |
| C19orf84 | 0.000427 | <0.05 |
| MYO9B | 0.009028 | <0.05 |
| BPHL | 5.57E-05 | <0.05 |
| ZNF683 | 0.000753 | <0.05 |
| ZAP70 | 0.007889 | <0.05 |
| JUP | 0.000132 | <0.05 |
| ALDH1L1 | 0.000328 | <0.05 |
| ZMYND15 | 0.029038 | <0.05 |
| APOBEC3D | 0.003584 | <0.05 |
| LRFN1 | 6.08E-09 | <0.05 |
| CFB | 2.76E-05 | <0.05 |
| FBXO21 | 2.43E-06 | <0.05 |
| APOL2 | 0.043399 | <0.05 |
| ZNF189 | 1.37E-06 | <0.05 |
| ATP11A | 1.54E-06 | <0.05 |
| PANK1 | 3.23E-08 | <0.05 |
| CCR4 | 0.016113 | <0.05 |
| APOL1 | 0.026843 | <0.05 |
| CD68 | 0.046981 | <0.05 |
| FKBP11 | 2.12E-09 | <0.05 |
| PELI2 | 9.81E-07 | <0.05 |
| UNC13D | 0.000506 | <0.05 |
| SLCO5A1 | 0.000519 | <0.05 |
| TMEM25 | 1.58E-06 | <0.05 |
| RTP5 | 0.025969 | <0.05 |
| PML | 0.004407 | <0.05 |
| TLN2 | 1.05E-08 | <0.05 |
| PRKD1 | 2.07E-05 | <0.05 |
| AGAP2 | 0.007635 | <0.05 |
| RELB | 0.008825 | <0.05 |
| ZNF80 | 9.90E-05 | <0.05 |
| BST2 | 0.013316 | <0.05 |
| ZHX3 | 0.001295 | <0.05 |
| PIM2 | 0.002668 | <0.05 |
| KAZN | 0.000429 | <0.05 |
| DPP9 | 0.002294 | <0.05 |
| CYP39A1 | 0.013737 | <0.05 |
| ZDHHC15 | 0.002889 | <0.05 |
| FCRL5 | 0.001504 | <0.05 |
| VAV3 | 3.38E-06 | <0.05 |
| ISG15 | 0.003971 | <0.05 |
| NEUROG3 | 0.002051 | <0.05 |
| ACAP1 | 0.000184 | <0.05 |
| MEI1 | 0.000354 | <0.05 |
| BHMT | 4.71E-05 | <0.05 |
| DERL3 | 4.78E-06 | <0.05 |
| KLRK1 | 0.002288 | <0.05 |
| RNASE4 | 0.010698 | <0.05 |
| KIR2DL4 | 0.002491 | <0.05 |
| IGLL5 | 0.014838 | <0.05 |
| ETNK2 | 0.000176 | <0.05 |
| RNASET2 | 2.04E-05 | <0.05 |
| MZB1 | 0.002415 | <0.05 |
| NTNG2 | 0.000727 | <0.05 |
| RAB37 | 0.042738 | <0.05 |
| MASP1 | 7.11E-05 | <0.05 |
| CHST9 | 0.024921 | <0.05 |
| C17orf107 | 0.000268 | <0.05 |
| CCL22 | 0.00307 | <0.05 |
| AQP4 | 0.001733 | <0.05 |
| VEPH1 | 0.003526 | <0.05 |
| RASSF8 | 0.005227 | <0.05 |
| TEX11 | 0.000498 | <0.05 |
| AJAP1 | 1.14E-07 | <0.05 |
| MAGEC3 | 0.00347 | <0.05 |
| C8G | 0.001592 | <0.05 |
| DARS1 | 8.66E-05 | <0.05 |
| TCF19 | 0.002036 | <0.05 |
| FMO4 | 0.004227 | <0.05 |
| CD5L | 0.035165 | <0.05 |
| DPEP1 | 0.003762 | <0.05 |
| CTSE | 0.002852 | <0.05 |
| JSRP1 | 1.59E-07 | <0.05 |
| OR13A1 | 5.47E-06 | <0.05 |
| GGACT | 0.007089 | <0.05 |
| PTGR1 | 0.000519 | <0.05 |
| ABCD1 | 0.022441 | <0.05 |
| STBD1 | 0.008671 | <0.05 |
| ARHGEF3 | 0.030293 | <0.05 |
| CASR | 0.0349 | <0.05 |
| RIPOR2 | 0.002453 | <0.05 |
| HSPA6 | 0.02627 | <0.05 |
| GALM | 0.001213 | <0.05 |
| CEACAM1 | 2.05E-06 | <0.05 |
| RAB29 | 0.000443 | <0.05 |
| PAEP | 1.43E-07 | <0.05 |
| SYTL1 | 0.000813 | <0.05 |
| SLC13A1 | 0.000609 | <0.05 |
| DOCK8 | 4.77E-06 | <0.05 |
