# Supplementary material for: Construction and validation of programmed cell death-based molecular clusters for prognostic and therapeutic significance of clear cell renal cell carcinoma
Source: Heliyon. 2023 May 2;9(5):e15693. doi: 10.1016/j.heliyon.2023.e15693 (PMC10256830; doi:10.1016/j.heliyon.2023.e15693)
Supplement: Multimedia component 1 [file mmc1.zip › Table S4.docx]

Table S4. Top 30 upregulated genes in cluster A and B.

| Gene | Cluster | Type |
| --- | --- | --- |
| FDCSP | A | Up |
| PAEP | A | Up |
| SAA2-SAA4 | A | Up |
| PAGE2B | A | Up |
| CRP | A | Up |
| MMP13 | A | Up |
| MAGEA10 | A | Up |
| ANGPTL8 | A | Up |
| ROS1 | A | Up |
| STMN2 | A | Up |
| EPYC | A | Up |
| SLC18A3 | A | Up |
| CALY | A | Up |
| LHX2 | A | Up |
| MAGEC3 | A | Up |
| GOLGA6L2 | A | Up |
| GOLGA6L7 | A | Up |
| KERA | A | Up |
| EDN3 | A | Up |
| NKX2-5 | A | Up |
| ANXA8 | A | Up |
| SCNN1G | A | Up |
| IVL | A | Up |
| IGLL5 | A | Up |
| TNNT1 | A | Up |
| LCN1 | A | Up |
| TRIML2 | A | Up |
| ZIC2 | A | Up |
| DMRT3 | A | Up |
| SAA4 | A | Up |
| ALB | B | Up |
| CYP17A1 | B | Up |
| UGT2A1 | B | Up |
| SLC13A2 | B | Up |
| SLC7A13 | B | Up |
| PLG | B | Up |
| CTXN3 | B | Up |
| SLC22A12 | B | Up |
| SLC22A8 | B | Up |
| SLC6A19 | B | Up |
| TMEM213 | B | Up |
| PAH | B | Up |
| TMEM174 | B | Up |
| ZNF804B | B | Up |
| CYP4A11 | B | Up |
| SLC22A6 | B | Up |
| CYP4A22 | B | Up |
| SLC34A1 | B | Up |
| AGXT | B | Up |
| PLPPR1 | B | Up |
| TACR3 | B | Up |
| LPA | B | Up |
| BMP5 | B | Up |
| CYP3A4 | B | Up |
| ARG1 | B | Up |
| CA4 | B | Up |
| SLC22A13 | B | Up |
| HMGCS2 | B | Up |
| NUPR2 | B | Up |
| ATP4B | B | Up |
