# Supplementary figures and images for: Construction and validation of programmed cell death-based molecular clusters for prognostic and therapeutic significance of clear cell renal cell carcinoma
Source: Heliyon. 2023 May 2;9(5):e15693. doi: 10.1016/j.heliyon.2023.e15693 (PMC10256830; doi:10.1016/j.heliyon.2023.e15693)

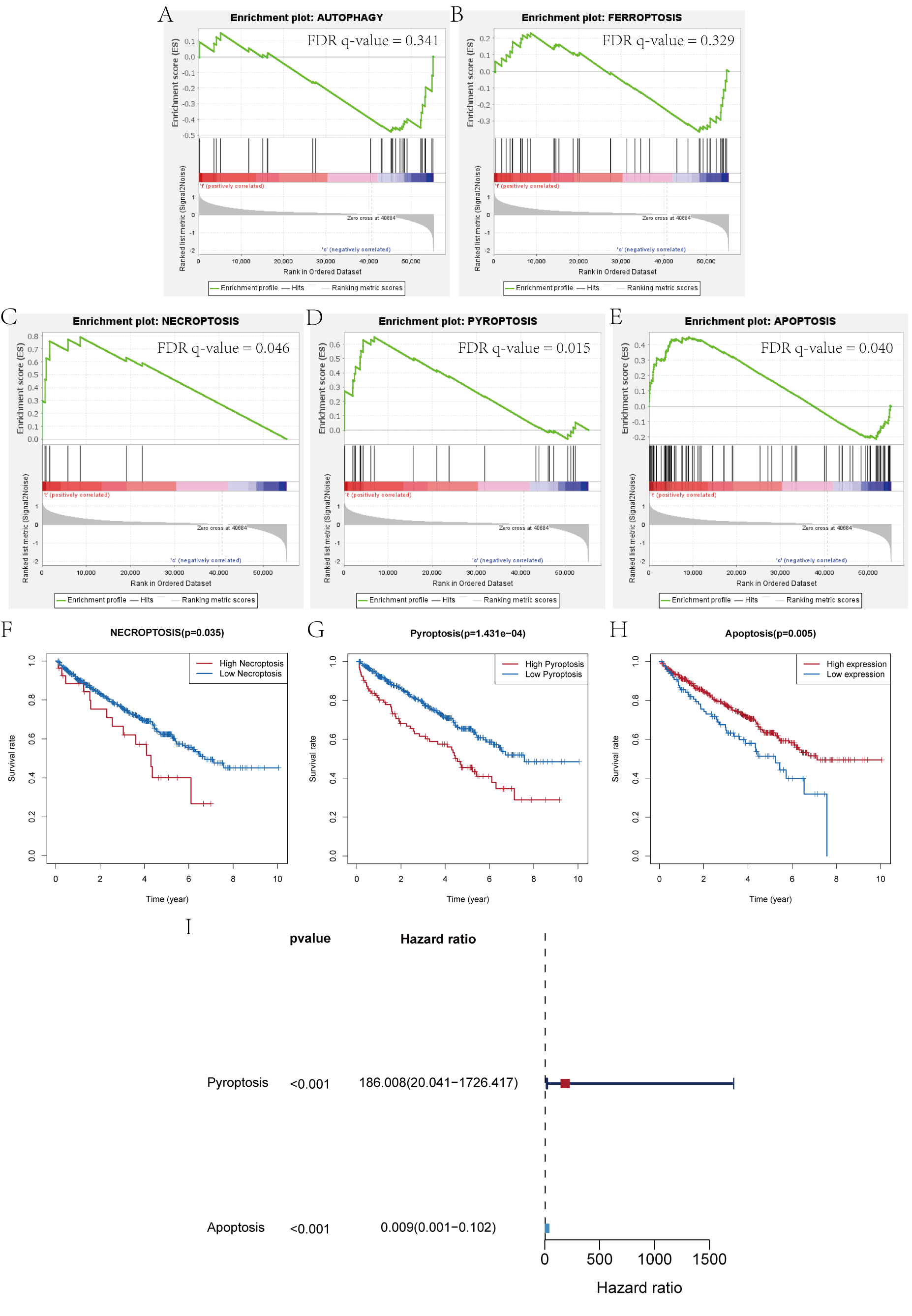

Supplement: Multimedia component 1 [file mmc1.zip › Figure S1.tif]

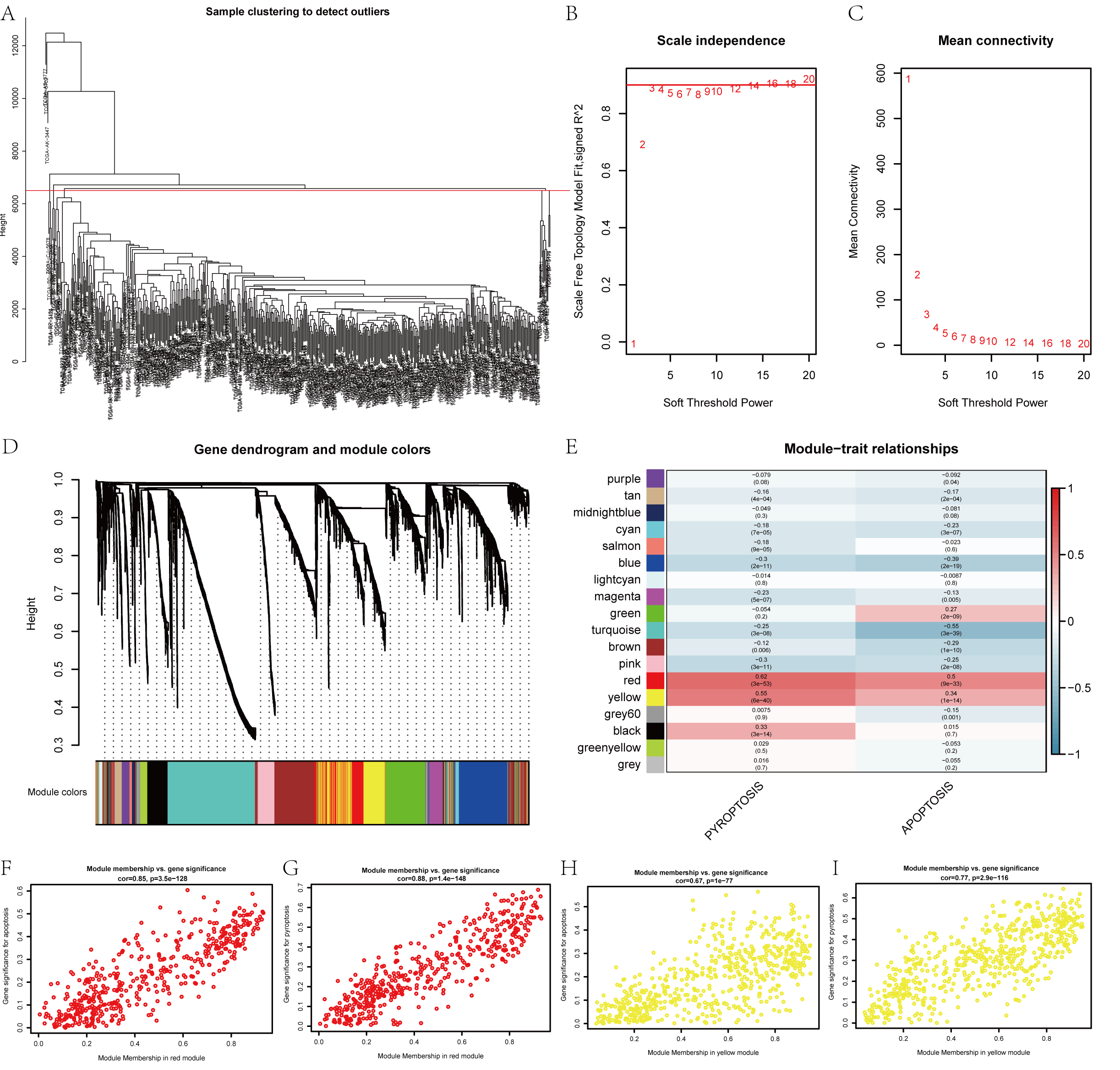

Supplement: Multimedia component 1 [file mmc1.zip › Figure S2.tif]

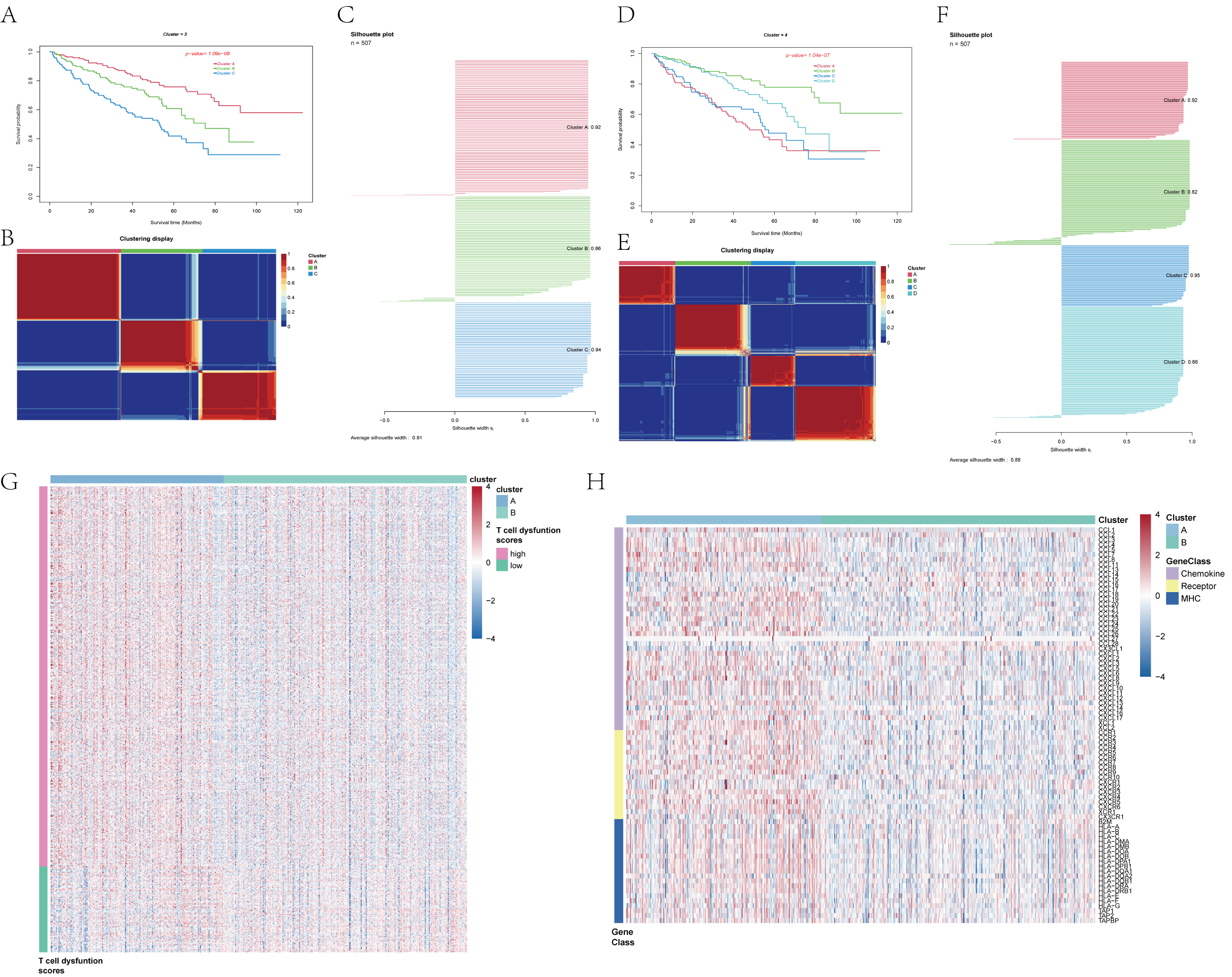

Supplement: Multimedia component 1 [file mmc1.zip › Figure S3.tif]

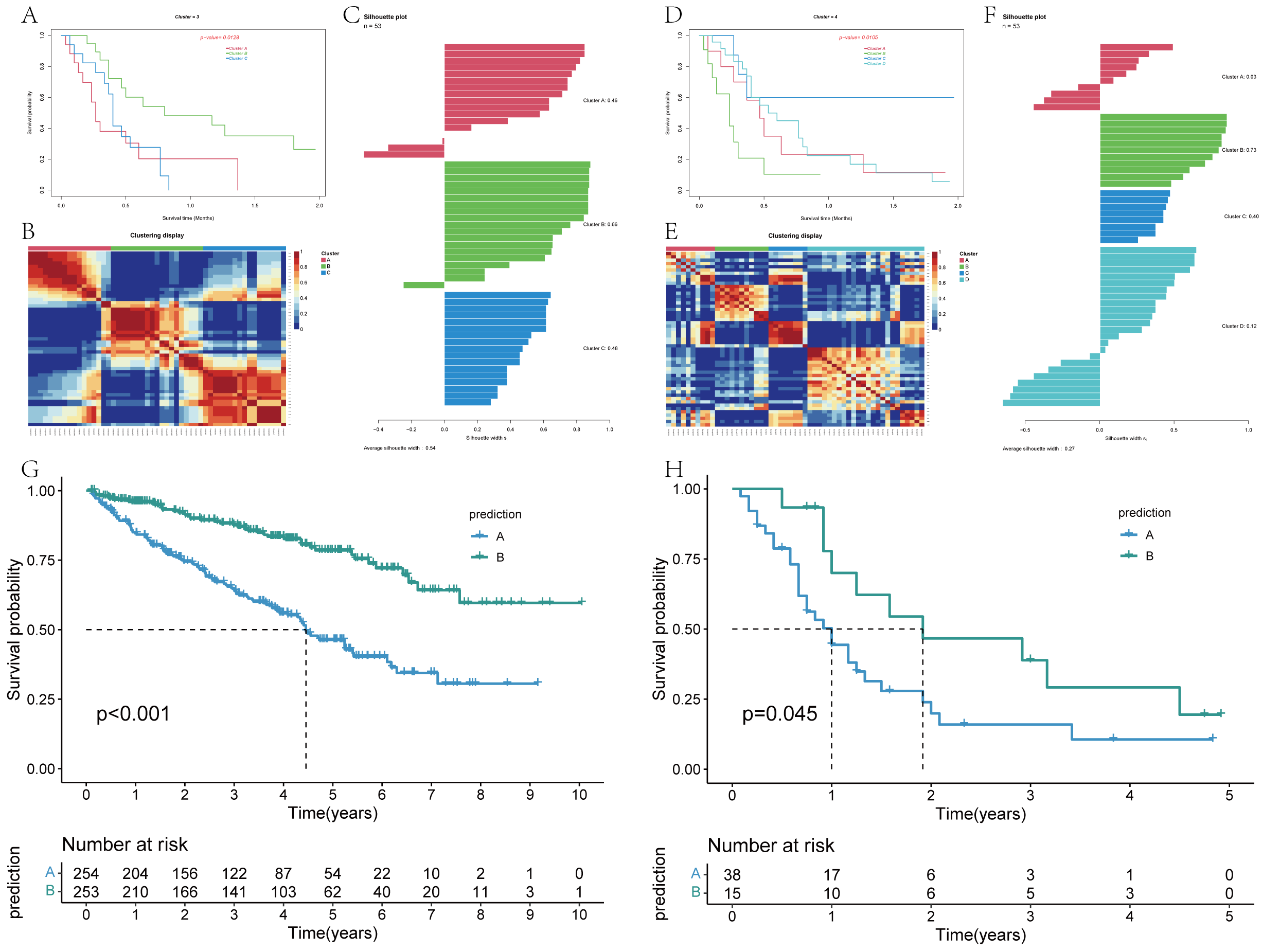

Supplement: Multimedia component 1 [file mmc1.zip › Figure S4.tif]
